# Supplementary material for: Relevance of Mediterranean diet as a nutritional strategy in diminishing COVID-19 risk: A systematic review
Source: PLoS One. 2024 Aug 21;19(8):e0301564. doi: 10.1371/journal.pone.0301564 (PMC11338465; doi:10.1371/journal.pone.0301564)
Supplement: Attachment — Submitted filename: revision-1.pdf [file pone.0301564.s005.pdf]

General comment:

The topic approached by authors of this paper is interesting and overall, I believe the study has relevant information hereby suitable for publication after revision.

The authors clearly explained their work throughout the manuscript. I just have a suggestion to point out.

The paper is generally well-written and easy to follow. However, certain sections, particularly the results and discussion, could benefit from more concise language and avoidance of repetition.

Specific comments:

Introduction:

- It could benefit from a more detailed explanation of the specific mechanisms through which the Mediterranean diet might influence COVID-19 outcomes.

- To strengthen the context, it would be good to include more recent studies and comprehensive statistics on the global impact of COVID-19.

Methods:

- Specific search terms and possible language restrictions could be included in the search strategy.

- More details on the scoring process of the Newcastle Ottawa Scale (NOS) and how the authors addressed any biases identified would enhance the reader's understanding of the robustness of the findings.

- The cross-sectional design of the studies included in this review limits the ability to establish causation.

- There is a limited number of studies included in this review.

Results:

- The narrative synthesis of the data is clear, but the inclusion of a meta-analysis could provide a more quantifiable measure of the impact of the Mediterranean diet.

Discussion:

- Further discussion on the biological plausibility of the Mediterranean diet’s effects on immune function and inflammation in the context of viral infections would be beneficial.
