# Supplementary material for: Relevance of Mediterranean diet as a nutritional strategy in diminishing COVID-19 risk: A systematic review
Source: PLoS One. 2024 Aug 21;19(8):e0301564. doi: 10.1371/journal.pone.0301564 (PMC11338465; doi:10.1371/journal.pone.0301564)
Supplement: Attachment — Submitted filename: Response to Reviewers.docx [file pone.0301564.s006.docx]

Response to Reviewers’ Comments

**Reviewer #1: General comment:**

- *The topic approached by authors of this paper is interesting and overall, I believe the study has relevant information hereby suitable for publication after revision.*
- *The authors clearly explained their work throughout the manuscript. I just have a suggestion to point out.*
- *The paper is generally well-written and easy to follow. However, certain sections, particularly the results and discussion, could benefit from more concise language and avoidance of repetition.*

Dear reviewer, we would like to thank you for your time and attention in reviewing the manuscript and giving your inputs. Your suggestion has been noted, and several changes and omission have been made in the Results and Discussion section to avoid repetition.

**Specific comments:**

- ***Introduction:***
- *It could benefit from a more detailed explanation of the specific mechanisms through which the Mediterranean diet might influence COVID-19 outcomes.*

Noted, we have added line 84-86 “Mediterranean diet is rich sources of polyphenols and polyunsaturated fatty acids (PUFAs), which are well-known antioxidants”, line 88-89 “…while also exerting antiviral effects by decreasing nuclear transcription factor kappa B (NF-κB) expression “, line 92-98 “Fruits and fish oil, both vital components of Mediterranean diet, contain vitamin A, C, and D. These vitamins are effective antioxidant against reactive oxygen species (ROS) which are secreted by immune cells and help maintain body cells integrity, while also supporting epithelial barriers integrity. Vitamin D, which stimulate polymorphonuclear (PMN) and natural killer cells (NK) cells in producing potent anti-microbial peptides. The resulting effect are decreased risk of infection and increased viral clearance”. We hope this addition will better explain the rationale in our systematic review.

- *To strengthen the context, it would be good to include more recent studies and comprehensive statistics on the global impact of COVID-19.*

Noted, we have added line 63-67 “COVID-19 severity largely played a role in COVID-19 mortality, as reflected from recent data that reported COVID-19 mortality ranged from 6.3% in average population, to 13.2% in inpatient population, and drastically increased to 55.9% in patients requiring mechanical ventilation. Until this article was written, more than 7 million total deaths by COVID-19 were recorded”. These data are sourced from recent studies and the latest update from the WHO website to provide better insight on COVID-19 statistics.

- ***Methods:***
- *Specific search terms and possible language restrictions could be included in the search strategy.*

The specific search terms, search strategy in detail, along with medical subject headings (MeSH), have been attached as Supplementary File 1 (S1 File). Language restriction is not applied in our search strategy, however, our reviewers excluded articles not available in English in the screening process.

- *More details on the scoring process of the Newcastle Ottawa Scale (NOS) and how the authors addressed any biases identified would enhance the reader's understanding of the robustness of the findings.*

Noted, we have added line 141-144 “NOS criteria were scored based on three categories: selection, comparability, and outcome, with each category consisted of 1-4 items. NOS score ranged from 0 (lowest quality) to 9 (highest quality)” to provide better details on the Newcastle Ottawa Scale scoring. Based on our scoring process, we identified one potential bias that seemed to stand out among others, as mentioned in strengths and limitations section line 326-329 “The major limitation was that Mediterranean diet adherence of all studies was self-reported. Measurement errors in FFQs are known to affect results of studies and may have biased the observed effect estimates. FFQs were prone to biased responses, such as recall and estimation bias.” We are aware of this potential bias, as self reported FFQs fell under the category of exposure in Newcastle Ottawa Scale, and five out of six studies were deducted a point because of this. However, the use of FFQs are justified in most studies since is realistic and is logistically feasible (line 329-330), especially with the high number of total patients and their varied diets. In addition to Newcastle Ottawa Scale, we also used the tool by Page et al. and RoBANS 2 to evaluate any reporting biases. Utilizing both of these tools, we obtained that the included studies were of low-to-moderate risk for reporting bias

- *The cross-sectional design of the studies included in this review limits the ability to establish causation.*
- *There is a limited number of studies included in this review.*

We concur, as mentioned in our discussion section, line 275-277, regarding the study by Zagarzadeh et al., “...the study had a cross-sectional design, and as stated by its authors, the results cannot infer a causative association” and the strengths and limitations section, line 339 “…there were few studies included in this systematic review.” These limitations need to be recognized and considered cautiously when interpreting the results.

- ***Results:***
- *The narrative synthesis of the data is clear, but the inclusion of a meta-analysis could provide a more quantifiable measure of the impact of the Mediterranean diet.*

We do acknowledge this limitation, as mentioned in our discussion section line 287-288 “Although a meta-analysis could produce a higher statistical power for this purpose, there were a few considerations as to why it was not performed.” However, we also mentioned several reasons for this, in the next few lines “Multiple assessment index for Mediterranean diet adherence were used between studies. In addition, most studies assigned points for Mediterranean diet adherence depending on sex-specific median consumption in the respective study. Hence, we deemed a meta-analysis was not appropriate for the diverse data.” We hope this would give a satisfactory explanation to the readers regarding our decision.

- **Discussion:**
- *Further discussion on the biological plausibility of the Mediterranean diet’s effects on immune function and inflammation in the context of viral infections would be beneficial.*

Noted, we have added line 299-302 “Vitamin C, a major vitamin in fruits and vegetables, took part in inhibiting NLRP3 inflammasome pathway, decreasing pro-inﬂammatory cytokines, and improving neutrophil chemotaxis. These resulted in minimizing viral pathogenesis, enhancing recovery, while also preventing respiratory viral infection”, line 305-309 “Docosahexaenoic acid (DHA) and eicosapentaenoic acid (EPA) bind to peroxisome proliferator-activated receptors (PPARs), inhibiting cytokine expression by NF-κB inflammatory transcription factor [38]. Other pathways include decreasing lipid rafts and augmenting major histocompatibility complex 1 (MHC 1) expression”, line 320-321 “…. inhibiting cyclooxygenase-1 (COX-1) and cyclooxygenase-1 (COX-2) enzyme expression”, and line 315-316 “…antioxidants which neutralize free oxygen radicals released by neutrophils, minimizing cellular damage”.

**Reviewer #2:**

*The manuscript is well written. In the introduction, the author explained the reason for the scientific gap very well. Also, the Material and Method section and the discussion are detailed and precise. Overall, well-conducted study.*

We would like to thank the reviewer for reviewing and complimenting the manuscript. Please do not hesitate to contact us for further queries.
